# Particle Pollution Estimation Based on Image Analysis

**DOI:** 10.1371/journal.pone.0145955

**Published:** 2016-02-01

**Authors:** Chenbin Liu, Francis Tsow, Yi Zou, Nongjian Tao

**Affiliations:** 1 School of Chemistry & Chemical Engineering, Nanjing University, Nanjing, JiangSu, China; 2 Center for Bioelectronics and Biosensors, Biodesign Institute, Arizona State University, Tempe, Arizona, United States of America; 3 Beijing Kinto Investment Management Co., Ltd, Beijing, China; Zhejiang Univ, CHINA

## Abstract

Exposure to fine particles can cause various diseases, and an easily accessible method to monitor the particles can help raise public awareness and reduce harmful exposures. Here we report a method to estimate PM air pollution based on analysis of a large number of outdoor images available for Beijing, Shanghai (China) and Phoenix (US). Six image features were extracted from the images, which were used, together with other relevant data, such as the position of the sun, date, time, geographic information and weather conditions, to predict PM_2.5_ index. The results demonstrate that the image analysis method provides good prediction of PM_2.5_ indexes, and different features have different significance levels in the prediction.

## Introduction

Among various air pollutants, airborne particulate matter (PM), especially fine particles with diameters less than 2.5 micrometers (PM_2.5_), has a huge adverse effect on human health [1], including increased rates of cardiovascular, respiratory and cerebrovascular diseases [2]. Various techniques have been developed to measure the mass concentrations of PM in air. The most popular methods include filter-based gravimetric methods [3], tapered element oscillating microbalance [4], beta attenuation monitoring [5], optical analysis [6,7] and black smoke measurement [8]. All these methods require sophisticated equipment, which is out of reach for most people. A simple, fast and cheap method to monitor PM in air have the potential to increase public awareness, alert those with respiratory diseases to take proper prevention measures, and provide local air quality data that are not otherwise available.

PM pollution is often characterized by poor visibility, arising from scattering of sunlight by airborne particles. A layperson can tell the difference between clear and hazy sky, but it is much more difficult to distinguish if the hazy sky is caused by PM or fog, and to quantify the degree of PM pollution. Digital cameras are widely available to provide high quality photos, which, together with the ever-increasing computational power for sophisticated image processing with even a mobile device, provide a new opportunity to qualify and analyze airborne particles based on digital photography. Wang et al. [9] examined air quality from light extinction estimated from photographs. However, airborne PM affects a photograph via complex scattering of light, depending on angle and intensity of sunlight, position and angle of the camera, distance between the objects and camera, as well as weather conditions, which are reflected in multiple ways: obscuring the images of distant objects, discoloring the sky and reducing the image contrast [10]. Accurate assessment of PM pollution requires us to consider multiple image features and image recording conditions.

Here we report a method to detect and quantify PM pollution by extracting a combination of six image features, including transmission, sky smoothness and color, whole image and local image contrast, and image entropy. We further consider the time, geographical location, and weather condition of each photo, to determine the correlation between PM level and various factors. Based on these features, we build a regression model to predict PM level using photos collected in three different cities, Beijing, Shanghai and Phoenix, about 1 year. Many of today’s smartphones are equipped with high quality imaging and powerful computing capabilities, which could be used to detect and quantify PM_2.5_ in air by analyzing the photographs of outdoor scenes.

We arrange the present paper in the following orders. First, the optical model of a hazy image formation was described. Second, according to the model analysis, several features were extracted from hazy images, and the support vector regression was applied to train and predict the PM index. Finally, we evaluate the performance and discuss possible ways to improve the accuracy of the present method.

## Principle

**Fig 1:**
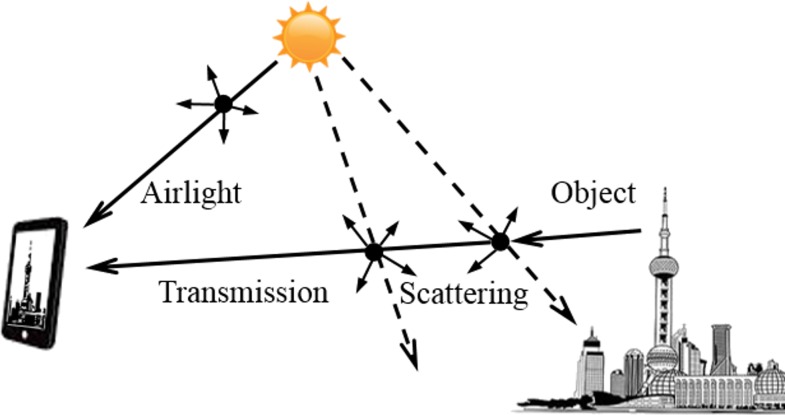
The radiance reaching the smartphone camera is the summation of the transmitted light from the object and airlight from the sun after scattering by air, water and PM in atmosphere.

PM in air affects an optical image in different ways, but they are all originated from the interactions of light with the airborne particles, mainly via light scattering, including Rayleigh scattering and Mie scattering [11]. Light scattering causes an attenuation of light transmission in air, which can be expressed by the Beer-Lambert law,

$$t=e^{-\beta d}$$ (1)

where *β* is the medium extinction coefficient, which depends on particle size and concentration, and *d* is the distance of light propagation. This equation indicates that if the extinction coefficients at different wavelengths are determined, then PM concentration can be estimated. The extinction coefficient may be determined from an observed image according to [1,12–14],

I(x,y)=t(x,y)J(x,y)+(1−t(x,y))A (2)

where *I* is the observed hazy image, *t* is the transmission from the scene to the camera, *J* is the scene radiance, *A* is the airlight color vector (see explanation below). As shown in Fig 1, the first term of Eq 2 is the direct transmission of the scene radiance into the camera, which is light reflected by the object surfaces in the scene and attenuated by air before entering the camera. The second term (1-*t*(*x*,*y*))*A* is called airlight, which is the ambient light scattered by air molecules and PM into the camera [12–15]. Wang et al. [9] applied the above formula to estimate light attenuation. In the present work, the relationship between transmission value and PM density was evaluated by analyzing ROIs at difference distances. Eq 2 assumes constant atmospheric and lighting conditions, which, in practice, may both change with the weather and position of the sun that vary with the time of the day and season. Additionally, both *J* and *A* depend not only on the weather and position of the sun, but also on PM distribution and concentration. The present work considers these varying factors as additional features to improve the accuracy of PM estimation based on images. Fig 1.

The above discussion did not consider color information explicitly, which can also serve as important features for PM estimation based on light scattering consideration. Rayleigh scattering dominates when the particles (including air molecules) are much smaller than the wavelength of light. It is strongly wavelength dependent, and varies with wavelength (λ) according to λ^-4^, which is responsible for the blue color of the sky. In contrast, Mie scattering occurs when the size of the particles is comparable to the wavelengths of light, which tends to produce a white glare around the sun when particles are present in air. The combination of Rayleigh and Mie scattering affect the brightness and color saturation of an outdoor image. Conversely, the color and brightness information contains particle concentration and size information, and can be used as distinct features to estimate PM. The present work includes color information as important image features for PM estimation, in addition to light attenuation.

## Materials and Method

### Data acquisition

**Fig 2:**
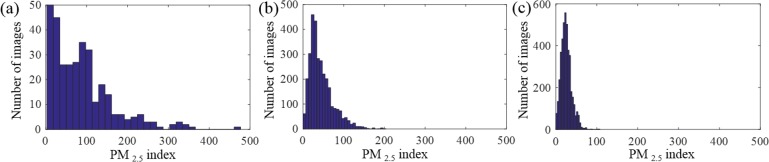
The histogram of PM_2.5_ in different cities. (a) Beijing; (b) Shanghai; (c) Phoenix.

To evaluate the capability and accuracy of PM estimation based on image analysis, it is critical to build a database. In the present work, we collected images, as well the date and time of each image, PM_2.5_ index, weather data and geographic location from fixed scenes in three cities, Beijing and Shanghai (China), and Phoenix (U.S.). The Beijing dataset consists of 327 photos (Taken by one of the co-author Yi Zou) of a fixed scene, featuring Beijing Television Tower, captured at almost the same time every morning in 2014. The Shanghai dataset contains 1954 photos of the Oriental Pearl Tower, the icon of Shanghai, from Archive of Many Outdoor Scenes (AMOS) dataset, captured every hour from 8:00 a.m. to 16:00 p.m., from May to December in 2014 [16]. The Phoenix dataset includes 4306 images from AMOS dataset [16], captured every half hour from 9:00 a.m. to 16:30 p.m. in 2014. The PM_2.5_ indices of Beijing and Shanghai were from published documents by the U.S. consulates, which monitor the air quality of the two cities. The air quality of Phoenix was from the published data by U.S. Environmental Protection Agency [17]. Fig 2 shows the PM_2.5_ index range in the three cities. The weather data of the three cities were obtained from Weather Underground (http://www.wunderground.com/) and Weather Spark (https://weatherspark.com/). Precise geographical locations, including longitude, latitude and altitude, were from Google map (https://www.google.com/maps) and elevation map (http://elevationmap.net). Fig 2. (a) Beijing; (b) Shanghai; (c) Phoenix.

### Method

**Fig 3:**
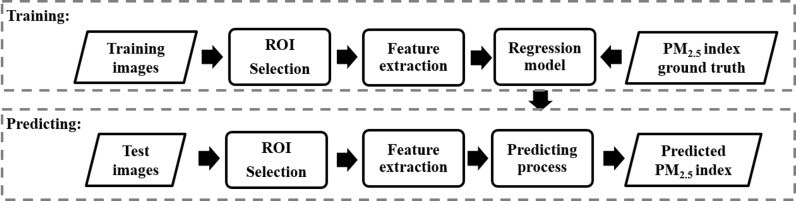
PM estimation via outdoor image analysis.

After building the database described above, we applied the following image processing algorithm to estimate PM_2.5_ index. As shown in Fig 3, the algorithm mainly consists of the following steps: regions of interest (ROI) selection, feature extraction, regression model training and predicting. We describe the details of these steps below. Fig 3.

#### ROI selection

**Fig 4:**
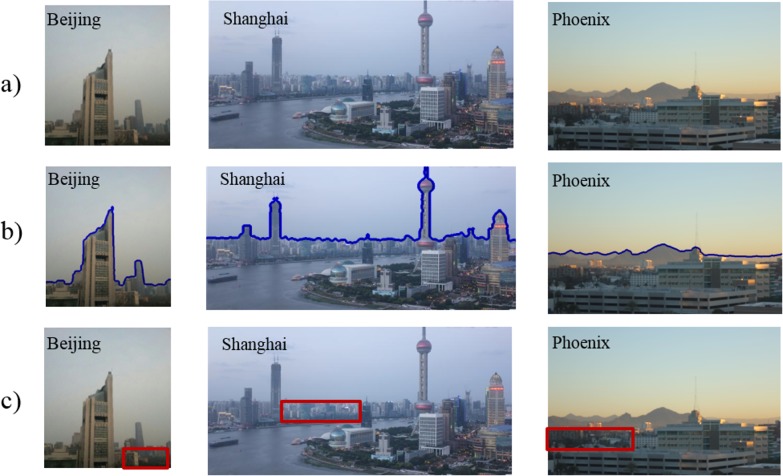
Sample photos in our haze detection database. a) Photos captured at Beijing, Shanghai and Phoenix respectively. b) Boundary lines (blue lines in b) between distant buildings and sky. c) Selected ROIs (red boxes). Reprinted under a CC BY license, with permission from [Yi Zou], original copyright [2014].

The first step is to remove the watermarks in these photos. The watermarks indicate the date and time stamp in our images, which appear in white characters in the first or last few rows. The second step is to build a mask of the sky region, which appears in the images of all the three cities. Fig 4A shows three representative images, one from each city. Both the buildings and background sky are clearly visible. The color images were converted into gray scale images, and then further into binary images with the Otsu method. The Otsu method converts gray scale to binary images by selecting a threshold that minimizes the intra-class variance or maximizing the inter-class variance [18]. In these images, the intensity of the sky is higher than that of the buildings, so the upper part of the binary image is mainly the sky. Fig 4B shows blue lines that mark the boundary between the sky and buildings. To remove the noise caused by the white buildings, we applied the opening operator with a 4×4 disk structuring element, and then filled the holes in the binary image. The third step is to draw the ROIs for the distant buildings manually as shown in Fig 4C, which were used to examine the transmission difference at different distances and PM densities. The ROIs were selected in one image in each dataset and applied to the rest. Fig 4. a) Photos captured at Beijing, Shanghai and Phoenix respectively. b) Boundary lines (blue lines in b) between distant buildings and sky. c) Selected ROIs (red boxes). Reprinted under a CC BY license, with permission from [Yi Zou], original copyright [2014].

#### Feature extraction

According to the model described in the principle section, transmission can be used to describe the attenuation of scene radiance. To solve for the transmission and thus the attenuation with a single hazy image, the concept of dark channel has been introduced, which assumes the existence of some pixels with zero or very low intensity at least for one color channel in all the outdoor images [13–16]. For a haze–free image *J*, the dark channel is,

$$J^{dark}(x)=\underset{y\in \Omega (x)}{\text{min}}(\underset{c\in \left\{r,g,b\right\}}{\text{min}}(J^{c}(y)))$$ (3)

where *J*^*c*^ is one of the color channels of *J*, and Ω(*x*) is a local patch centered at *x*. The airlight can be estimated from the sky or the brightest region, so the transmission can be obtained by,

$$\tilde{t}(x)=1-\underset{y\in \Omega (x)}{\text{min}}(\underset{c\in \left\{r,g,b\right\}}{\text{min}}\frac{I^{c}(y)}{A})$$ (4)

where *I*^*c*^(*y*)∕*A* is the hazy image normalized by air-light A, and the second term on the right is the dark channel of the normalized hazy image.

**Fig 5:**
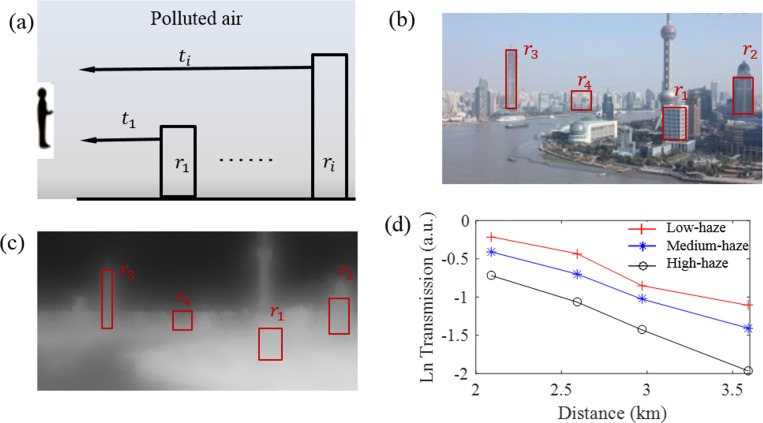
The transmission decreases as the distance or PM_2.5_ index increases. (a) Schematic illustration of transmission variation with distance. (b) Four ROIs (r_1_~r_4_) located at increasing distances. (c) The estimated transmission map. (d) Semi-logarithmic plots of transmission curves vs. distance under different haze conditions.

An important assumption in the present model is that the transmission decreases exponentially with the distance between the object in the scene and the camera. We evaluated the transmission by analyzing images of objects at different distances (Fig 5A). Fig 5B shows four ROIs (marked by red boxes) for buildings located at different distances from the camera (r_1_<r_2_<r_3_<r_4_). The transmission map (Fig 5C) shows the four ROIs at different distances. The average transmission values obtained for the four ROIs are plotted in a semi-logarithmic scale, showing exponential decrease of the transmission with distance, which confirms the validity of the Beer-Lambert law. Fig 5. (a) Schematic illustration of transmission variation with distance. (b) Four ROIs (r_1_~r_4_) located at increasing distances. (c) The estimated transmission map. (d) Semi-logarithmic plots of transmission curves vs. distance under different haze conditions.

Image contrast is another feature related to PM concentration in air. In fact, human visual perception of air quality is related to image contrast, or visibility [19, 20]. The effect of PM on image contrast can be understood based on Eq 2. As PM concentration increases, the airlight term (second term of Eq 2) arising from light scattering by PM increases. Airlight does not contain information of the scene, which leads to a decrease in the image contrast due to PM. Because transmission decreases with the distance between an object and camera, the airlight term contribution also increases with the distance, so the higher is the PM concentration, the lower is the image contrast.

There are many ways to quantify the contrast of an image. A simple way is to use the root mean square (RMS) of an image to describe image contrast. This approach has been found to match with human perception of image contrast [21]. RMS contrast is defined as the standard deviation of the image pixel intensities,

$$RMS=\sqrt{\frac{1}{MN}\sum_{i=1}^{N}\sum_{j=1}^{M}{\left(I_{ij}-avg(I)\right)}^{2}}$$ (5)

where *I*_*ij*_ is intensity at (*i*,*j*) pixel of the image with size *M* by *N*, and *avg*(*I*) is the average intensity of all pixels in the image. RMS contrast does not depend on the spatial frequency content, nor convey any information about the spatial distribution of image contrast.

Another image feature that can possibly provide PM information is image entropy, which quantifies information contained in an image, and is related to image texture. Image entropy is defined as,

$$entropy=-\sum_{i=1}^{M}p_{i}\;{\text{log}}_{2}\;p_{i}$$ (6)

where *p*_i_ is the probability that the pixel intensity is equal to *i*, and *M* is the maximum intensity of the image. As the PM concentration increases, the image increasingly loses its details, and the image entropy decreases.

**Fig 6:**
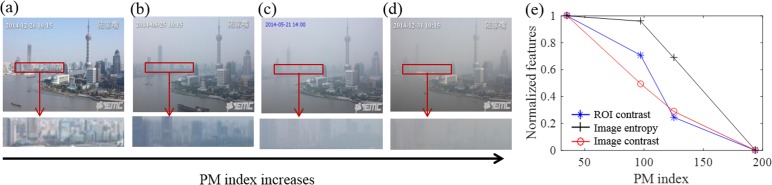
Image features variation with PM index. (a~d): Hazy images showing that the contrast of the building region decreases with the PM index, where the lower panel shows the zooming-in images of the regions marked by the red boxes. (e) The normalized features vs. PM_2.5_ index plot, including ROI RMS contrast (blue), image entropy (black), and image RMS contrast (red).

To determine the image contrast and entropy, we first converted a color images into a gray scale image, and then calculated the image entropy and RMS contrast for the entire image. For comparison, we also determined image contrast of distant buildings by calculating RMS of the selected ROI. Fig 6A–6D show the image of the Oriental Pearl Tower of Shanghai recorded on different days with increasing PM_2.5_. As the PM_2.5_ level increases, the visibility deceases, which is especially clear in a zoomed-in region (red box). The RMS contrast and image entropy both decreases with PM_2.5_ index (Fig 6E). Note that the RMS values of the entire image and the ROI show similar results.

Sky region also carries useful information, such as weather condition. Due to light scattering, the sky is blue on a clear day and gray or white on a hazy or cloudy day. The presence of cloud in the sky can be directly detected from the image, which can be used to differentiate it from the hazy sky. By combining the color and smoothness features, we have attempted to determine clear, partly cloudy, cloudy and hazy days. This information, together with the online weather data, help minimize errors in the estimation of PM due to weather conditions.

**Fig 7:**
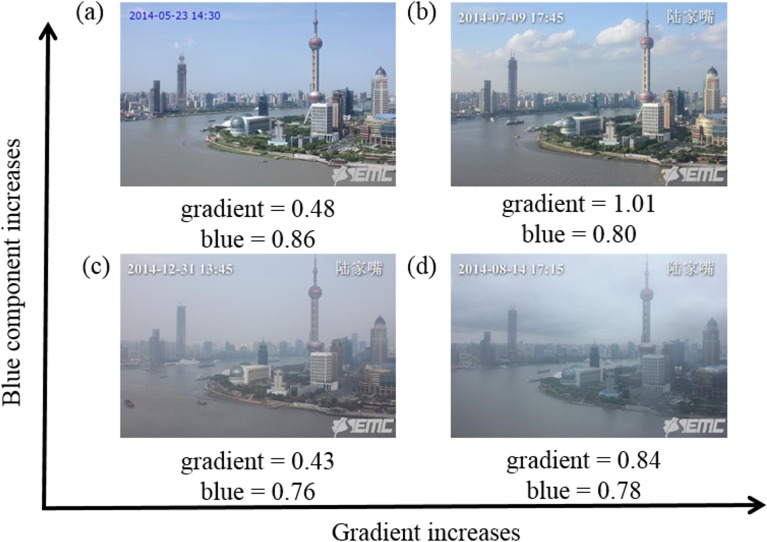
Sky gradient and blue component provide weather information, such as cloud formation. (a) Sunny day; (b) Partly cloudy/sunny day; (c) Hazy day; (d) Cloudy day.

The color of the sky in our study is presented by the average value of the blue component of RGB channels in sky region. The blue channel and sky mask were used to extract the blue component of the sky image (Fig 7). The average of the blue component describes the color of the sky. Fig 7. (a) Sunny day; (b) Partly cloudy/sunny day; (c) Hazy day; (d) Cloudy day.

The smoothness of the sky is defined by the average of the gradient amplitude in the sky region. The image gradient is defined as,

$$\nabla I=\frac{\partial I}{\partial x}\hat{x}+\frac{\partial I}{\partial y}\hat{y}$$ (7)

where *I* is the intensity of the sky region in the image, ∂*I*/∂*x* is the gradient in *x* direction, and ∂*I*/∂*y* is the gradient in *y* direction. The average of the gradient amplitude is defined as,

$$avg(\left|\nabla I\right|)=\frac{1}{MN}\sum_{i=1}^{N}\sum_{j=1}^{M}\sqrt{{\left(\frac{\partial I}{\partial x}\right)}^{2}+{\left(\frac{\partial I}{\partial y}\right)}^{2}}$$ (8)

where *avg*(*) is the average value of the two-dimensional image of size *M* by *N*. As shown in Fig 7, the blue component in sunny and partly cloudy images is higher than those in the cloudy and hazy images. The averages of the gradient amplitude in the sky regions are higher in cloudy day images than those in sunny and hazy day images.

**Fig 8:**
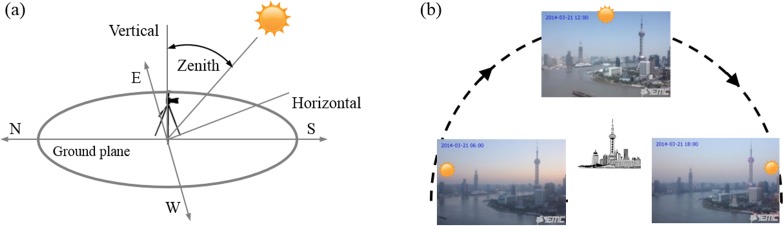
Sky color dependence on solar zenith angle. (a) Definition of solar zenith angle. (b) Sample images show that the sky near horizon is red during sunrise and sunset on the same day compared with noon time.

Both Rayleigh and Mie scattering depend strongly on the angle of sunlight reaching the object, which is determined by the position of the sun for a given scene (Fig 8A). For example, different angles produce different images, such as sunrise and sunset shown in Fig 8B. In the present study, both the scenes and cameras are fixed in positions, so the variation of the scattering is mainly due to the angle of incident illumination, which is determined by the position of the sun. Fig 8. (a) Definition of solar zenith angle. (b) Sample images show that the sky near horizon is red during sunrise and sunset on the same day compared with noon time.

The zenith angle of the sun as shown in Fig 8A indicates the elevation of the sun above the horizon. It is a function of the observer local time, date, longitude, latitude and altitude. In our study, we used solar position algorithm to calculate the solar angle [22]. The main steps include calculations of (1) the earth heliocentric longitude, latitude, and radius with local information, (2) the geocentric longitude, latitude and the aberration correction, (3) the Greenwich apparent sun longitude and sidereal times, (4) the geocentric sun declination and observer local hour angle, and (5) the topo-centric sun position and solar angle. The solar zenith angle is defined as,

$$\theta_{s}=\text{arccos}(\text{sin}\;\varphi \;\text{sin}\;\delta +\text{cos}\;\varphi \;\text{cos}\;\delta \;\text{cosh})$$ (9)

where *φ* is the local latitude, *δ* is the sun declination, and h is the local hour angle. In this study, we obtained the longitude, latitude, altitude, coordinated universal time, local date and local time of the captured image. This information is available online, and can also be obtained from the built-in GPS and gyroscope features of smartphones.

#### Support vector regression

After defining the image features that are possibly related to PM concentration, we determine the relationship between the extracted features and PM concentration using nonlinear support vector machine and kernel to predict the PM concentration. Support vector machines (SVM) [23] have been widely applied in a large number of fields [24–26], including prediction and regression [27, 28]. SVM can also be used to solve nonlinear regression estimation problems, called support vector regression (SVR). In this paper, we used SVR to predict PM_2.5_ index.

The basic idea of SVR is to map input data to a higher dimensional feature space via a function, Φ. A linear function f in the high dimensional feature space formulates nonlinear relationship between input and output data. The regression function can be expressed as,

f(w,b)=w⋅Φ(x)+b (10)

where *f*(*w*,*b*) is the forecasting values, *w* and *b* are the function parameter vectors, and Φ is a nonlinear transformation from *x* to high-dimensional space. The goal of SVR is to minimize function,

$$R_{reg}(f)=\frac{1}{N}\sum_{i=1}^{N}\Theta_{\varepsilon }(y_{i},w^{T}\Phi (x)+b)$$ (11)

where Θ_ε_ is the ε-insensitive loss function and defined as,

$$\Theta_{\varepsilon }(y,f(x))=\{\begin{array}{c} \left|f(x)-y\right|-\varepsilon ,if\left|f(x)-y\right|\geq \varepsilon \\ 0,if\left|f(x)-y\right|<\varepsilon \end{array}$$ (12)

where ε is a measure of training error, called the radius of the insensitive tube.

In addition, Θ_ε_ is used to determine the optimal hyper plane in the high dimensional space and minimize the training error between the input data and the ε-insensitive loss function. Then, SVR minimizes the overall errors,

$${\text{min}}_{w,b,\xi^{*},\xi }(\frac{1}{2}w^{T}w)+C\sum_{i=1}^{N}\left(\xi_{i}^{*}+\xi_{i}\right)$$ (13)

with the constraints, *y*_*i*_-(*w∙*Φ(*x*)+*b*)≤*ε*+*ξ*_*i*_, (*w∙*Φ(*x*)+*b*)- *y*_*i*_≤*ε*+*ξ*_i_^*^, *ξ*_i_^*^,*ξ*_*i*_≥0, *i* = 1,2,…,N, where *ξ*_*i*_ and *ξ*_i_^*^ are slack variables, and *C* is the cost constant. The training vector *x*_*i*_ are mapped to a higher dimensional space with Φ. The radial basis function (RBF) kernel is a popular kernel function used in regression and classification, which can handle the nonlinear relationship well, which is defined as,

$$K(x_{i},y_{j})=\text{exp}(-\gamma {\left|x_{i}-x_{j}\right|}^{2})$$ (14)

where γ is a kernel parameter. The parameters that dominate SVR are the cost constant, *C*, and kernel parameter, γ. We performed grid search [28] to determine the optimal values of *C* and γ (*C* = 2^8^, γ = 2^2^). We used the toolbox LIBSVM in MATLAB R2013a and 2-fold cross validation as the regression strategy. We have also performed leave one out cross validation, and found similar results.

## Results and Discussion

**Fig 9:**
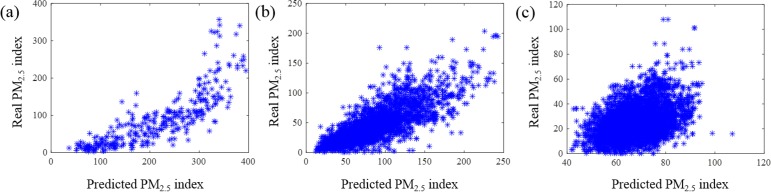
Real PM_2.5_ index vs. predicted PM_2.5_ index plot. (a) Beijing; (b) Shanghai; (c) Phoenix.

To predict the PM_2.5_ index with the regression model, we randomly selected half of the samples as training data, and the other half as the testing data, and then we considered the second half as training data and first half as the testing data. For each city’s data, we used the 2-fold cross validation and obtained the prediction results. Fig 9 plots the real PM_2.5_ index vs. predicted PM_2.5_ index. The prediction error was evaluated with root mean square error (RMSE), R-squared and F-test. RMSE is defined as,

$$RMSE=\sqrt{\frac{1}{N}\sum_{i=1}^{N}{\left(y_{i}-{\hat{y}}_{i}\right)}^{2}}$$ (15)

where ${\hat{y}}_{i}$ is the i^th^ forecast value, and *y*_*i*_ is the i^th^ observed value, *i* = 1,2,…,N. R-squared is given by

$$R^{2}=1-\frac{\sum_{i=1}^{N}{\left(y_{i}-{\hat{y}}_{i}\right)}^{2}}{\sum_{i=1}^{N}{\left(y_{i}-avg(y)\right)}^{2}}$$ (16)

where ${\hat{y}}_{i}$ is the i^th^ forecast value, *avg*(*y*) is the average value, *y*_*i*_ is the i^th^ observed value, *i* = 1,2,…,N. R-squared increases with the agreement between the model prediction and actual result with a maximum value of 1, corresponding to perfect match of the prediction and the actual result. F-test evaluates the null hypothesis that all regression coefficients are equal to zero vs. the alternative that at least one does not. A significant F-test indicates that the observed R-squared is reliable. Fig 9. (a) Beijing; (b) Shanghai; (c) Phoenix.

Fig 9 shows that the predictions for the Beijing and Shanghai data correlate well with the actual PM_2.5_ indices. In contrast, the correlation for Phoenix is less obvious, which is mainly caused by that PM_2.5_ index in Phoenix falls within a narrow range (0–40). The PM_2.5_ indices in Beijing and Shanghai can reach as high as 340 and 204, respectively, while the PM_2.5_ index in the Phoenix can only reach 38, far below those in Beijing and Shanghai. Table 1 lists RMSE values, which shows that RMSE in Beijing is larger than that in Shanghai, which is caused by the prediction error for high PM_2.5_ index data (over 120), as shown in Fig 9A. R-squared values in Beijing and Shanghai are better than that in Phoenix due to the reason discussed above. F-test in Table 1 shows that R-squared values are reliable.

**Table 1:** Assessment of the support vector regression.

dataset	RMSE	R squared	F test
Beijing	42.69	0.65	p<0.0001
Shanghai	19.23	0.57	p<0.0001
Phoenix	2.89	0.22	p<0.0001

### Weather conditions

In the present work, the effect of different weather conditions was also taken into consideration in PM_2.5_ index prediction. Rainy and snowing days were rare in these datasets, we thus focused on two weather conditions: clear and cloudy days. In the Beijing dataset, 139 and 181 photos were captured on clear and cloudy days, respectively. In the Shanghai dataset, 548 and 1275 photos were captured on clear and cloudy days, respectively. For each weather condition and city, we used the same regression method and 2-fold cross validation as described above.

As shown in Table 2, the prediction error on cloudy days is larger than that in clear days. This is mainly caused by the water droplets in the air, which also scatter light. High humidity can significantly increase the effect of air pollution on visibility. For example, PM attracts water molecules leading to hygroscopic growth in ambient atmosphere [29]. When relative humidity reaches 80%, particles can grow to sizes that cause large increase in light scattering [30].

**Table 2:** Regression results for different weather conditions.

dataset	RMSE		R squared		F test	
	Clear	Cloudy	Clear	Cloudy	Clear	Cloudy
Beijing	38.90	58.52	0.55	0.45	p<0.0001	p<0.0001
Shanghai	13.11	25.18	0.58	0.48	p<0.0001	p<0.0001

Since the relationships between particle concentration, relative humidity and visibility are complicated, relative humidity was added as a feature to build the regression model in the following test. Results with and without humidity taken into account as one of the features are shown in Table 3. We can see that the prediction improved after adding the humidity feature for both datasets, especially in the case of Shanghai. This observation correlates with the fact that there are more images captured on cloudy days than that on clear days in Shanghai than in Beijing, and also Shanghai is usually more humid than Beijing. Considering that many of today’s newer smartphones are incorporating humidity sensors (e.g. Samsung S4), it is possible to include the humidity as a key feature to estimate PM_2.5_ index.

**Table 3:** Regression results with and without humidity as a feature.

dataset	RMSE		R squared		F test	
	Without	With	Without	With	Without	With
Beijing	42.69	40.43	0.65	0.68	p<0.0001	p<0.0001
Shanghai	19.23	14.05	0.57	0.72	p<0.0001	p<0.0001

### Feature assessment

To evaluate the features used in the current method, we calculated the distance correlation (DC) and Pearson correlation (PC) between each extracted feature and PM_2.5_ index. The distance correlation is a measurement of dependence between random vectors. DC varies between 0 and 1, representing low and high correlation between an extracted feature and PM_2.5_ index. PC is a measure of linear relationship between two vectors, which varies from -1, indicating a perfect negative linear relationship, to 1, indicating a perfect positive linear relationship.

As shown in Table 4, the transmission has one of the largest correlations with PM_2.5_ index, which is an important indicator of PM concentration. The ROI contrast, whole image contrast and image entropy also show good correlations with PM_2.5_ index, supporting human visual perception that the visibility decreases with increasing PM_2.5_ index. The sky smoothness and color analysis have some correlations with PM_2.5_ index. As for solar zenith angle, the DC and PC values are low, indicating little correlation of the quantity with PM_2.5_ index. The statistical analysis shows that transmission, image contrast and sky features are good features for PM_2.5_ estimation. We also calculated the PC between solar angle and other features. The DC and PC between solar zenith angle and sky smoothness are 0.38 and 0.22 respectively, and the DC and PC between solar zenith angle and sky color are 0.24 and 0.23 respectively. The solar angle shows correlations with sky smoothness and color. The normalization of the related image features with the solar zenith angle could improve the prediction accuracy.

**Table 4:** The features and their correlations with PM_2.5_ index in our dataset.

Features	Beijing		Shanghai		Phoenix	
	DC	PC	DC	PC	DC	PC
Transmission	0.81	-0.78	0.60	-0.60	0.32	-0.32
ROI contrast	0.82	-0.76	0.40	-0.40	0.28	-0.29
Image entropy	0.63	-0.54	0.42	-0.46	0.24	-0.24
Image contrast	0.43	-0.43	0.52	-0.55	0.13	-0.12
Sky smoothness	0.34	-0.29	0.32	-0.31	0.28	-0.30
Sky color	0.43	-0.43	0.20	-0.21	0.09	-0.08
Solar zenith angle	0.13	-0.01	0.12	-0.11	0.10	-0.04

**Fig 10:**
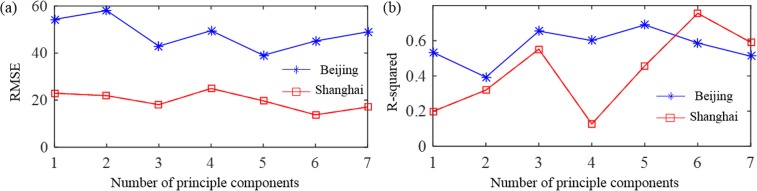
PCA-SVR results for Beijing and Shanghai’s dataset. (a) RMSE. (b) R-squared.

In this study, two feature optimization methods were used to evaluate the feature redundancy and achieve the optimized regression performance. The first one is principle component analysis (PCA) [31]. The second one is sequential backward feature selection (SBFS) [32], and the criterion is RMSE. All eight features were included in the datasets: transmission, ROI contrast, image entropy, image contrast, sky smoothness, sky color, solar zenith angle and relative humidity. We used PCA on the datasets to reduce the dimensions, and then perform the training and regression with 1~7 principle components (PC) respectively. The PCA-SVR results are shown in Fig 10. Compared with the previous results, the regression error in Beijing’s dataset (RMSE: 39.08, R squared: 0.69) is smaller using the first 5 PCs. For Shanghai’s dataset, the regression performance is also better using the first 6 PCs than that without PCA optimization (RMSE: 13.76, R squared: 0.76). From the above results, we can see that with more information, by including combined image features, weather and geographic information, the PM_2.5_ index prediction can be improved. To optimize the feature combination, the PCA-SVR method is efficient to reduce the computation burden and improve the PM_2.5_ index prediction. Fig 10. (a) RMSE. (b) R-squared.

To search for an optimal feature subset, SBFS method was used. Considering there are 8 features in the initial dataset, the method mainly includes: (1) remove the 1^st^ feature in the dataset *D*_8_, and obtain the regression error *e*_81_; (2) remove the i^th^ feature in *D*_8_, and obtain the regression error *e*_8i_; (3) repeat the above process, so we can get the errors {*e*_81_,*e*_82_,…,*e*_88_}; (4) the minimum error *e*_8j_ in {*e*_81_,*e*_82_,…,*e*_88_} corresponds to the optimized feature subset of 7 dimensions, called *D*_7_, and the j^th^ feature is considered as the least important one in *D*_8_; (5) remove the feature sequentially and repeat steps (1)~(4), so we can get the feature subsets, {*D*_1_,*D*_2_,…,*D*_8_}, which are considered the optimized feature subset for each dimension; (6) in these feature subsets {*D*_1_,*D*_2_,…,*D*_8_}, the optimal subset is the one with the minimized regression error. In Beijing and Shanghai’s datasets, the optimal features include transmission, ROI contrast, image entropy and sky smoothness. Shanghai has a humid subtropical climate, thus, the relative humidity is one of the most important features. From the regression errors, we can see the feature selection method improves the prediction accuracy. Both PCA and SBFS methods can reduce the feature redundancy and improve the regression with comparable regression errors (Table 5). The PCA method converts the original features into PCs with orthogonal transformation, so PCs not more than the original number of variables can be chosen to be used in the PM_2.5_ index prediction. SBFS method selects an optimal feature subset in the original feature space, so we can improve our understanding in feature contribution and potentially develop new features based on the optimal subset.

**Table 5:** The performance comparison between all the features and the optimized feature subsets for Beijing and Shanghai’s dataset.

Dataset	All the features		PCA		SBFS	
	RMSE	R squared	RMSE	R squared	RMSE	R squared
Beijing	40.43	0.68	39.08	0.69	38.28	0.70
Shanghai	14.05	0.72	13.76	0.76	13.65	0.76

The method presented here has several limitations that may be improved in the future. 1) More image features can be included and analyzed, and an optimal combination of different features can be developed with genetic algorithms or particle swarm optimization methods [33], 2) better algorithm, such as deep convolutional neural network can take advantage of the two-dimensional structure of an input image and be used to perform machine learning task, 3) Additional information, such as magnetometer, gyroscope, and thermometer, could be used to determine the camera angle and meteorological parameters.

## Conclusions

We have developed an image-based method to estimate PM_2.5_ index in air. We have extracted various image features, including transmission, image contrast and entropy, sky smoothness and color, and studied their correlations with the reported PM_2.5_ indices in Beijing, Shanghai and Phoenix. We have also examined the effects of solar zenith angle, and weather conditions on the accuracy of the predictions. Using the image and non-image features, we have analyzed a large number of images captured in Beijing (327 images, one per day for 327 days), Shanghai (1954 images, and 8 images per day for 245 days), and Phoenix (4306 images, and 16 images per day for 270 days), and concluded that the method can provide reasonable prediction of PM_2.5_ index over a wide PM_2.5_ index range. We do not expect that the present method will replace the gold standard particle counting apparatus, however, its simplicity and smartphone readiness can help promote air pollution awareness, and alert people with serious respiratory diseases to stay away from suspected polluted air.
